# The physics-AI dialogue in drug design†

**DOI:** 10.1039/d4md00869c

**Published:** 2025-01-23

**Authors:** Pablo Andrés Vargas-Rosales, Amedeo Caflisch

**Affiliations:** a Department of Biochemistry, University of Zurich Winterthurerstrasse 190 8057 Zürich Switzerland caflisch@bioc.uzh.ch

## Abstract

A long path has led from the determination of the first protein structure in 1960 to the recent breakthroughs in protein science. Protein structure prediction and design methodologies based on machine learning (ML) have been recognized with the 2024 Nobel prize in Chemistry, but they would not have been possible without previous work and the input of many domain scientists. Challenges remain in the application of ML tools for the prediction of structural ensembles and their usage within the software pipelines for structure determination by crystallography or cryogenic electron microscopy. In the drug discovery workflow, ML techniques are being used in diverse areas such as scoring of docked poses, or the generation of molecular descriptors. As the ML techniques become more widespread, novel applications emerge which can profit from the large amounts of data available. Nevertheless, it is essential to balance the potential advantages against the environmental costs of ML deployment to decide if and when it is best to apply it. For hit to lead optimization ML tools can efficiently interpolate between compounds in large chemical series but free energy calculations by molecular dynamics simulations seem to be superior for designing novel derivatives. Importantly, the potential complementarity and/or synergism of physics-based methods (*e.g.*, force field-based simulation models) and data-hungry ML techniques is growing strongly. Current ML methods have evolved from decades of research. It is now necessary for biologists, physicists, and computer scientists to fully understand advantages and limitations of ML techniques to ensure that the complementarity of physics-based methods and ML tools can be fully exploited for drug design.

## Abstract 2

What are the potential impacts and limitations of machine learning models for ligand identification and optimization? We review the field and compare data-hungry models with physics-based methods.

## Graphical abstract

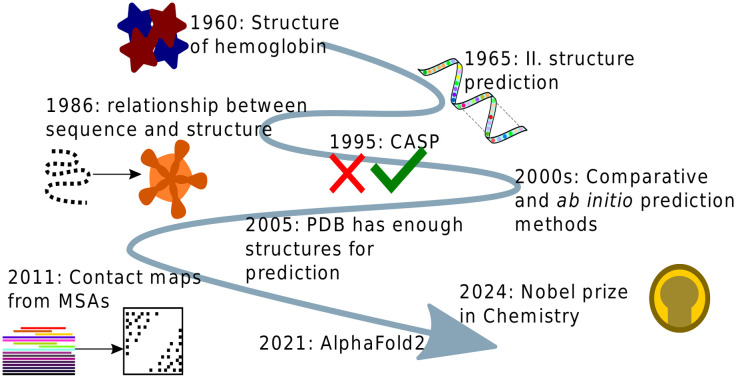

## 1. Introduction

### 1.1 The path to protein structure prediction

**Fig. 1:**
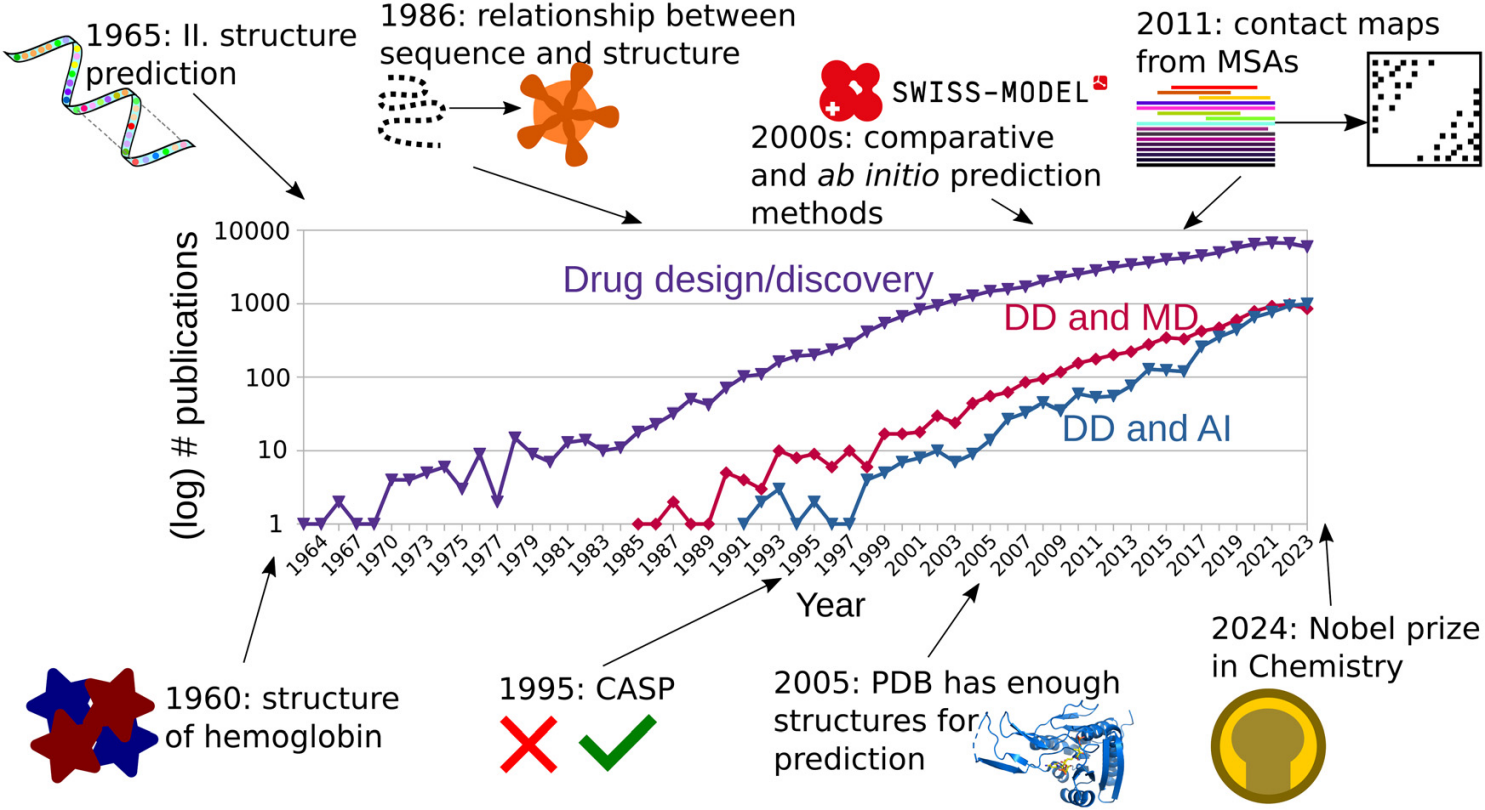
The number of publications mentioning drug design or drug discovery (DD) has continually increased since the 1960s (violet curve). The discovery of the structure of hemoglobin in 1960 opened the door to structure-based drug design. In the 1990s, the number of DD publications that mentioned MD simulations (red curve) or AI tools (blue curve) started to grow steadily. Since the late 2010s, the gap of DD publications based on AI methods *versus* MD simulations has narrowed.

More than 60 years ago, the first protein structures were determined experimentally. The three-dimensional conformations of myoglobin and hemoglobin were described by scientists at Cambridge and appeared published in 1960.^1,2^ First hand accounts of this momentous event help us understand the difficulty and work that went into these discoveries which today are routine work.^3–5^ This set the course for the beginning of the structural biology revolution and protein structure-based drug design (Fig. 1). Nevertheless, most protein structures remained unknown and biochemical analyses were the main method to obtain information about protein function and behavior. In 1961, Anfinsen *et al.*, showed that a ribonuclease could be reversibly denatured, and regain function after renaturing.^6^ Levinthal *et al.*, proceeded in similar way using alkaline phosphatase, from *Escherichia coli* and *Serratia marescens*, and not only found they could obtain active enzymes after renaturing them, but also that the interspecific dimer of the two was active as well. They therefore theorized that both must share a conserved active site, and a configuration which allows for active heterodimers.^7^ Thanks to the advances in the availability of protein sequence information, Perutz *et al.* proposed in 1965 that despite poor sequence conservation, the structure of globins was similar across all vertebrates.^8^ The foundational advances of the first half of the 1960 decade enabled Guzzo to postulate in 1965 that there was enough evidence that in proteins, “sequence implies structure”, and a thermodynamically most stable form must be the native and active one.^9^ These were the first building blocks for the successful protein structure prediction methods of today.

Already in the 1960s scientists tried to predict the structure of proteins from their sequence. In his paper, Guzzo did not only task himself with the understanding of protein function and its relationship to structure, but mostly with the prediction of protein structure.^9^ Due to the complexity of the task, he focused instead on predicting the secondary structure of proteins, with the hope that solving a smaller part of the problem might prove easier than predicting the whole tertiary structure, while still giving valuable insight into the fold of the protein. He analyzed the sequences and structures of myoglobin and the α- and β-hemoglobin to predict that α helices are disrupted by “critical residues”: P, D, E. and H. He later applied this prediction to TMV capsid and lysozyme.^9^ Such prediction was later expanded by Prothero, who gave more complex and complete rules on the influence of residues to secondary structure.^10^ The turn of the decade brought further research, incorporating more data, and more advanced analyses. Pain and Robson proposed a new approach in 1970, where pairs of residues were screened to understand the “helix-forming power” of each residue. They had the advantage of having more structures available.^11^ It was not until 1973 that Nagano proposed a statistical analysis based on 95 available proteins to predict helices, but also loops and *β*-strands. They did not only focus on pairs of adjacent residues, but also recognized the long-range influence of other residues away from the position considered.^12^ These years saw many more attempts at the prediction of protein secondary structure, with varying levels of success.^13,14^ Ten more years would still pass, until enough structures were available to generate a detailed and unambiguous definition of secondary structure.^15^

Since protein sequence encodes structure,^9,16^ it was theorized that the sequences of an entire protein family may also contain information about its tertiary structure.^17^ Pazos *et al.*^18^ showed in 1997 that the evolution of a protein must be, in some way, constrained by the sequence of that of its interaction partners, and these correlated mutations could be discovered in multiple sequence alignments (MSA). In the end they used this information to predict the interfaces between interacting proteins.^18^ Afterwards, Fariselli *et al.* used neural networks to predict the contact maps from a database of 173 proteins, with at least 15 sequences in each MSA. They obtained a relatively low accuracy, albeit the best for its time.^19^ Hopf *et al.* continued this line of work, using the higher number of sequences available 20 years later.^20,21^ In 2011, Morcos *et al.* used direct-coupling analysis to predict contact maps from MSAs.^22^ Coevolution methods continued to ripen in the decade of 2010 with new methodologies and proposed applications at the level of structures and interfaces, but also others such as binding site prediction.^23,24^

### 1.2 Comparative and *ab initio* modelling

A huge step in the protein prediction history can be traced to the introduction of the critical assessment of methods of protein structure prediction (CASP)^25^ in 1995. The standardized experiments, occurring every two years, started yielding information about the main bottlenecks in protein prediction.^26^ One category of such prediction methods was the *ab initio* method, where protein sequence is the main input for the prediction. *Ab initio* modelling usually follows physical principles, using techniques such as Monte Carlo sampling,^27,28^ threading (fold recognition),^29^ fragment based prediction,^30^ or stepwise secondary structure and then fold prediction.^31,32^ ROSETTA started as an *ab initio* prediction software based on the assembly of small fragments. It managed root mean square deviations of around 6 to 4 Å with respect to the native structure in CASP3.^33^ Distance based prediction methods also showed promise, when scientists realized that a subset of native inter-Cα distances could be used as additional restraints to generate native-like conformations using *ab initio* methods.^34,35^

Furthermore, the genomic explosion of the early 2000s generated a huge gap between the number of sequenced genes and their solved structures. For all the proteins for which their genetic sequences were known in 2004, only around 1% had their structure experimentally determined.^36^ Nevertheless, already since the 1980s, and thanks to previous observations such as those by Levinthal *et al.*,^7^ it had been proposed that there exists a strong relationship between sequence identity and fold conservation.^37^ This is why homology (or comparative) modelling also surged as an important category of protein structure prediction for proteins with known sequence but yet undetermined structure. Homology modelling usually consists of the following steps: identification of a template based on evolutionary closeness, alignment of target and template, modelling of conserved regions, modelling of divergent regions, assignment of sidechain rotamers, and refinement.^38^ For targets with strong evolutionary relationship to the templates, model building was usually simple except for nonconserved regions and loops, while more distant evolutionary relationships required more advanced alignment methods for finding templates.^39^ Early homology methods were able to correctly align models to templates with sequence similarities above 50%, while quality deteriorated for sequence identities lower than 35%.^40^ Despite their poor performance for distantly related proteins, comparative methods were pioneering in the use of evolutionary data at large scale. These homology modelling programs incorporated a combination of MSAs and structural methods to improve the alignment methods.^41^ One of the pioneering automated servers for homology modelling is SWISS-MODEL,^42^ which is still in use today.^43^ The current engine of SWISS-MODEL, ProMod3, implements homology modelling, as well as the loop and sidechain reconstruction, and inclusion of ligands in the binding pocket.^44^ A stand-alone program for comparative modelling is MODELLER, which implements all the steps of homology modelling described above. Unlike SWISS-MODEL, MODELLER is based on a probabilistic description of spatial restraints which guide the structural prediction.^45^ The alignment of templates is used to generate a probabilistic density function on which the template is aligned.^46^ The modelling of loops is achieved through a combination of structural restraints, the probabilistic information from the alignment, and force field information.^47^ This serves as an example that, as the years passed, the division between *ab initio* and comparative (data-driven) methods started to dilute.^48^ Another example of the fusion of different modelling methods comes from TASSER, where threading was used for template identification and followed by refinement.^49^ The increase in protein structures deposited in the PDB led Zhang and Skolnick to declare in 2005 that the protein folding problem could be solved based on the entries available at the time, given efficient fold recognition algorithms that could be used to assign templates to the sequence being predicted.^50^ The following years continued with incremental advances being reported for *ab initio* and comparative methods at the successive CASPs, albeit at a more modest pace than before,^51–53^ until the introduction of the first AlphaFold model at CASP13.^54,55^ Today, artificial intelligence (AI) and deep learning (DL) based methods for protein structure prediction and design are widely regarded as a revolution in life sciences, to the point that we talk of structural biology in terms of the times “before and after AlphaFold2”.^56^ The main developers of AlphaFold, John Jumper and Demis Hassabis, have shared the 2024 Nobel award in Chemistry with David Baker who has pioneered computational protein design (Fig. 1).

## 2. AI applications in structure prediction

### 2.1 Deep learning-based prediction

Today, after the explosion of machine learning (ML) in the 2010s, computer and biological scientists alike have worked to transfer the advances of AI, creating data-driven methods for protein structure prediction with a high level of success.^57^ The dream of scientists from the early 2000s of achieving proteome-scale prediction, uncovering new protein folds, and helping predict new functions,^58,59^ was finally achieved in 2023 (ref. 60) thanks to the establishment of the AlphaFold Protein Structure Database.^61^ It has been claimed that neural networks seem to have “largely solved” the protein folding problem at the domain level,^62^ although as Bowman correctly points, fields are advanced, not solved.^63^ Such claims can derive from a lack of understanding of the background of AlphaFold, and the work behind it which explains how it achieved such remarkable accuracy at the prediction of tertiary structure. Recapitulating some points discussed above, the biochemical studies of 1960s elucidated the structural similarity of proteins related by evolution. This was exploited by scientists who tried to predict contacts based on evolutionary information, the ones who tried to model novel structures based on already available structures of homologous proteins, or the ones who tried to recognize folds from primary sequence. In parallel, decades of experimental structure determination yielded a database of globular proteins which is complete enough for an advanced data mining strategy to take advantage of it. The success of AlphaFold2 can then be understood as the result of an excellent fold recognition procedure, which exploits the completeness of the library of single-domain proteins in the PDB.^64^ Therefore this revolution did not occur in isolation, half a century of research paved the way for the AI-based methods of protein structure prediction and design.

A common feature of all the AlphaFold models,^55,65,66^ and indeed of other related methods such as RosettaFold,^67,68^ is the use of MSAs to find evolutionary relationships that can be used to predict inter-residue contacts.^22^ The first AlphaFold model used the MSAs to bias a statistical potential of inter-residue interactions^69^ that better satisfies these contacts.^55^ AlphaFold2 uses the transformer architecture^70^ to integrate the information from the MSA and structural templates together.^65^ The MSA is so important to AlphaFold2, that it has been pointed out that AlphaFold2 has learnt a MSA–structure relationship, not a sequence–structure relationship as claimed.^71^ AlphaFold3 still uses the MSA to find information on close-by residue pairs, but the MSA is not used as input to the network directly.^66^ The MSA is completely ignored in ESMFold, a language model which captures evolutionary relatedness by learning dependencies between aminoacids at the sequence level, and then using this information to predict the contact map.^72^ The architecture of the ML models also reflects the directions of research in the deep learning community in general. While the transformer is present in the AlphaFold2 and ESMFold structure modules,^73^ AlphaFold3 and RosettFoldAllAtom use diffusion models to generate the final structures.^66,68^ Diffusion models are increasingly being used in structural biology and drug discovery,^74^ finding applications in protein design,^75,76^ conformer generation,^77^ and small molecule binder design.^78^

### 2.2 The challenges of DL-based methods

Despite the claims of protein structure prediction being “solved”,^63^ several challenges remain. Even some globular proteins can be predicted incorrectly,^79^ and extreme care must be taken when using AI structure prediction tools for disordered proteins.^80^ For example, AlphaFold2 overestimates the confidence of structure predictions in thousands of intrinsically disordered regions that fold upon binding or modification.^81^ Several strategies have been implemented to make “safer” predictions of these disordered regions. Bret *et al.* used fragments of disordered regions and different MSA schemes to predict interfaces of interacting disordered proteins.^82^ In recent work, we combined AlphaFold predictions of amyloid *β* dimers with molecular dynamics (MD) simulations to validate the predicted structures.^83^

Another important challenge in protein structure prediction is the generation of conformational ensembles. The prediction of single structures has been pointed as the current main limitation of these models.^84^ The generation of multiple structural models has been achieved in AlphaFold2 for example by activating dropout layers during prediction,^85^ subsampling the MSAs,^86,87^ MSA subsampling in combination with enhanced-sampling MD,^88^*in silico* mutagenesis of the MSA,^89^ flow matching,^90^ and others.^91^ Novel diffusion models are also emerging which are able to generate conformational ensembles, even of novel proteins.^92^ Finally, Cfold is an implementation of an architecture similar to AlphaFold, which was specifically trained with different conformations of the same sequence.^93^

A deeper understanding of the physics behind these models is also crucial to make the most of AI-powered protein structure prediction. Outeiral *et al.* found that the AI-based models are not appropriate tools to investigate folding, as the folding pathways they produce are inconsistent with experimental data.^94^ For fold-switching proteins it was found that AlphaFold2 assumes a “most-probable” fold while missing the other. Additionally, the chosen fold is predicted with an overestimated confidence due to the high conservation of these proteins.^95^ Later research theorized that prevalence of a single conformation is due to a memorization of the structures in its training set and not due to learning of a biophysical energy function. This renders the models unable to predict alternate conformations even in the presence of their binding partners.^96^ Indeed, it was shown that the performance of AlphaFold suffers with proteins that adopt diverse conformations.^97^ Such evidence is in clear contrast with previous claims that AlphaFold has learned an approximate biophysical energy function.^98,99^ It has been proposed that while AlphaFold and related methods learn the contacts between residues at the minimum of the free energy funnel characteristic of globular proteins, the shallow or multi-funneled landscapes of disordered and fold switching proteins counter this principle.^100^ A recent study in which perturbations were introduced to binding sites showed that AlphaFold3 does not predict binding based on molecular interactions, but based on general protein patterns. Thus non-physical predictions are possible because of overfitting to specific subsets of structural data.^101^ It is important to point out that the study was limited by the fact that AlphaFold3 was only available as a web server with limited capability for small molecule prediction. The fully open access to these deep learning models is not only essential to use them efficiently, but also to find new ways to improve them.

## 3. AI and the physics-based methods

### 3.1 Small molecule docking

Two physics-based methods essential to protein structure-based ligand design are MD simulations,^102–104^ and small molecule docking.^105–110^ Docking relies on scoring functions to describe protein–ligand interactions. The scoring functions have been classified into three main categories: force field-based, empirical, and knowledge-based.^111^ Force fields are analytical functions that make use of classical physics approximations of the potential energy of (macro)molecules which is calculated by the sum of bonding and non-bonding (van der Waals and Coulomb energy) contributions.^112^ The bonding interactions are calculated for pairs of atoms separated by one, two, or three covalent bonds, *e.g.*, Hooke's law is employed for the covalent bonds which does not allow the rupture of bonds or formation of new ones. The parameters of the force field are derived either from quantum mechanical calculations (*e.g.*, the partial charges for the Coulomb term) or by fitting to experimental data.^113,114^ The force fields might also include some desolvation terms, usually based on an implicit representation of solvent effects.^115–117^ For docking large libraries of compounds, the binding free energy is usually approximated by the difference between the energy of the protein/ligand complex and the energy of the unbound protein and ligand. Most frequently, the flexibility of the protein is ignored and entropic effects are neglected or approximated coarsely. Force field-based energy functions include those available in the docking programs SEED^118^ and AutoDock Vina.^119^ An additional sub-category, which is related to the force field-family, is the use of quantum mechanical descriptors for scoring. One example is the use of quantum mechanical “probes” which approximate a subset of the polar groups in the binding pocket of the target protein.^120^ Zhou and one of us screened a large library of compounds by the interaction energy with the probes calculated at a semi-empirical level of theory. In this way a novel and selective low micromolar inhibitor of the EphB4 tyrosine kinase was identified from a large library of compounds.^120^ Quantum mechanics-based scoring methods are less approximated than classical force fields, but are computationally more expensive.^121–123^

Unlike force field-based scoring functions, empirical scoring functions approximate the binding affinity directly. Analogous to force fields, they contain individual interaction descriptors of binding, trained using a regression model to fit the descriptors to the experimental binding affinity. Such descriptors can include intermolecular interactions like van der Waals and Coulomb terms, electrostatic desolvation penalty, ligand entropy and torsion, *etc.*^124^ Glide^125^ and ChemScore^126^ are examples of empirical scoring functions. Empirical scoring functions can also be employed for positioning small molecules in electron density maps determined by cryogenic electron microscopy (CryoEM).^127^

Knowledge-based scoring functions calculate the frequency of occurrence of the diverse atom pairs in a database from which, using the inverse Boltzmann relation, they obtain an approximation of the potential of mean force.^69,116^ An example of this type of functions is DrugScore.^128^ Interestingly, the concept of predicting and minimizing the potential of pairwise interactions was used as the basis of the first AlphaFold model,^55^ while the idea itself was already published in 1990.^129^ Due to the rapid changes and the different hybrid forms, Liu and Wang proposed in 2015 a new classification of scoring functions: physics-based methods (force field and quantum mechanics), empirical scoring functions, knowledge-based potentials, and descriptor-based scoring functions (such as those derived from ML).^130^

ML methods are becoming frequent in diverse aspects of scoring functions, and have performed well even when using simple methods such as a random forest.^131^ Two examples of ML-based scoring functions are PointVS^132^ and GNina.^133^ Guedes *et al.* have used linear and nonlinear ML methods to fit the coefficients of the physics-based terms of DockTScore, an empirical scoring function.^134^ Fujimoto *et al.* used molecular fingerprints of the protein–ligand interactions to build a regression model to approximate the potential mean force.^135^ As mentioned above, AlphaFold also incorporates concepts from knowledge-based scoring to predict protein structure.^55^ While not a knowledge-based model *per se*, Isert *et al.* used deep learning and quantum mechanics hand in hand for predicting protein–ligand binding affinity from CryoEM maps, giving strong emphasis to the study of interatomic interactions.^136^ Indeed with the deep learning explosion, came new and more data-hungry methods which unfortunately do not necessarily perform better than simpler “traditional” ML methods.^137,138^ Some of these deep learning methods were actually found to be even worse at generalizing than traditional docking methods.^139^ An important factor to keep in mind is that many ML scoring functions are applied at a postprocessing stage, with only select ones (such as GNina) being integrated into docking workflows.^140^ The use of different paradigms for sampling poses of the ligand and scoring them is not optimal. As an example, a force field-based sampling engine might not reach protein/ligand structures close to poses with optimal ML-based scores. The co-folding of proteins with their binding partners, for example as proposed by AlphaFold3, uses ML for both posing and scoring but can be affected by overfitting.^101^

### 3.2 Simulating the motion of atoms

Simulations are another important technique in drug design. They yield insights on the time-resolved behavior of biomolecules on an atomic scale. There are several types of atomistic simulations, such as Monte Carlo,^141^ MD,^142^ and quantum mechanics calculations.^143^ Monte Carlo simulations make use of random perturbations for iteratively evolving a molecular system. They can sample a thermodynamic ensemble but usually do not preserve the kinetic properties. In contrast, MD simulations are based on the classical Newtonian equations of motion (solved numerically) and thus not only reproduce a thermodynamic ensemble but also correctly reproduce the kinetics.^142^ Quantum mechanical simulations solve a system's electronic structure. This means they are very accurate and can describe processes such as chemical reactions, but are too slow to be applied to whole systems.^144^ Therefore, they are usually employed in combination with MD as multiscale simulations.^145^ MD has been used since long to obtain thermodynamics and kinetics of small molecule binding to proteins, validate predicted binding modes, to generate conformations for docking, identification of cryptic pockets, or (relative) binding free energy calculations.^146,147^ Simulations have been used to study the folding pathway of the cellular prion protein, from which druggable pockets were identified and targeted using small molecules to arrest folding.^148^ In another translational study, umbrella sampling MD simulations were successfully employed to predict the relative binding free energy of a series of anti-prion compounds which were then validated *in vivo*.^149^ Many simulation studies have been launched by different groups to analyze the self-assembly process of amyloid (poly)peptides.^150^ MD has also been used to try to open new avenues of treatment for amyloid diseases, by subjecting either small amyloidogenic fragments,^151^ or dimers of A*β*42 (ref. 83) to external electric fields. In our group, we have used MD and quantum mechanics (semi-empirical level) simulations to propose a catalytic mechanism for the human methyltransferase METTL3.^152^ We have also used MD to find structural information about binders for which no bound structure could be determined experimentally.^153^

MD simulations offer a means to evaluate the interaction free energy between a small-molecule ligand and its protein target, and rank ligands by relative affinity.^154^ In contrast to docking, MD simulations can take into account the full flexibility of the protein target, ligand, and surrounding solvent. They make use of the full force field, *i.e.*, including the bonded terms which is essential for reproducing the strain in the ligand upon binding. Free energy methods usually rely on a thermodynamic cycle to calculate the free energy differences between the states of interest. Since the direct calculation of the transformation of interest is usually difficult to obtain, a series of transformations is constructed that yields the same energy difference through simpler calculations.^155^ Two alchemical transformation protocols which can be used for free energy calculations are thermodynamic integration and free energy perturbation.^156–158^ Thermodynamic integration calculates the free energy difference between two states by numerically integrating the thermodynamic path between them. The path corresponds to an interpolation between the two end states' Hamiltonians, and is controlled by a coupling parameter.^159^ Free energy perturbation^160^ is based on the conversion of a molecule to another passing through unphysical intermediates of the two molecules.^161^ Instead of direct integration, the differences between small steps is used.^155^ If the systems are carefully prepared, which is time consuming and requires an in-depth knowledge of simulation protocols, free energy perturbation calculations yield an accuracy almost comparable to experimental measurement errors for relative binding free energy determination.^162,163^ Some challenges faced by these methods are the accuracy of the force fields used (which results in a systematic error) and the convergence of sampling (statistical error).^154,155^ Thermodynamic integration and free energy perturbation can be used for calculating both relative and absolute binding free energies.^164^ They have diverse applications in drug design, such as derivatization of ligands, scaffold-hopping, and binding pose validation.^165^ It is also possible to perform binding free energy calculations by Monte Carlo simulations in implicit solvent, using a thermodynamic cycle between the complex and the free protein and ligand. In a recent study, Monte Carlo sampling in implicit solvent with explicit ions as competitors, and the integration over multiple protonation states of protein and ligand, were assessed as a tool for virtual screening, and for the ranking of derivatives of hits obtained by docking.^166^

Several challenges remain in biomolecular modelling.^167^ One challenge is improving the physical models behind the simulations such as adjusting force fields to better represent disordered proteins^114^ or nucleic acids,^168^ in particular RNA.^169,170^ A second challenge of simulations is the timescales that can be reached. Even with recent advances in computing hardware, all-atom simulations remain prohibitive beyond the microsecond timescale. One possible solution is to leverage multiscale simulations to explore larger conformational spaces.^145,171,172^ Another interesting simulation protocol is enhanced sampling,^173,174^ for example by using swarms of trajectories to rebuild a reaction coordinate,^113,175,176^ or by using a(n approximate) reaction coordinate to reseed trajectories in a diverse manner.^177^ A good source of enhanced sampling protocols is PLUMED. The PLUMED library is a modular, open-source initiative which provides algorithms for enhanced-sampling MD, free energy methods, and analysis tools. Finally, steps are also being taken to optimize the different simulation packages to take advantage of current hardware architectures such as GPUs.^178,179^ A third challenge of biomolecular simulation is the integration of experimental data into the simulations.^167^ These integrative approaches incorporate data from different sources to understand biomolecules.^180^ Experimental data such as NMR or CryoEM has been used in conjunction with MD to understand RNA conformational diversity and dynamics.^181^ In the case of enhanced sampling protein simulations, it is very important to validate the obtained data against experimental data, due to the bias introduced.^182^ An example of an integrative modelling approach is metainference,^183^ which allows the construction of an ensemble of models consistent with experimental data by introducing the measurements as part of the energy function of the system.^184^

ML is also entering the world of biomolecular simulations. In the area of enhanced sampling, ML techniques have been applied to calculate reaction coordinates or collective variables for biased sampling.^185^ Designing or learning these reaction coordinates is difficult, and the simulation must be biased to sample the Boltzmann distribution appropriately. A more efficient approach would be to sample directly from the Boltzmann distribution to obtain the different conformations of the system. This is the idea behind Boltzmann generators. Boltzmann generators use neural networks to learn a transformation from a normal to a Boltzmann distribution, such that sampling from the normal can be used to generate many independent Boltzmann-distributed samples. Unlike enhanced sampling, they are not dependent on trajectory-based methods such as long simulations to obtain the samples.^186^ Another way to convert between distributions is flow matching, which has been used together with AlphaFold and ESMFold to predict protein ensembles. Similar to Boltzmann generators, flow matching uses a generative neural network to approximately transform a prior distribution to a Boltzmann one. Jing *et al.* proposes then to change AlphaFold from a regression model into a generative one, by feeding it with the noisy conformation generated by sampling from the prior and converting it to an approximately Boltzmann-distributed conformation. AlphaFold then “denoises” these generated coordinates and produces a high quality model based on this sample.^90^ In the force field development area ML applications have been extensively reviewed by Unke *et al.* and Chen *et al.*^187,188^ An example of a deep learning force field is shown by Majewski *et al.* They used MD data to construct an ML coarse-grained force field to recreate protein dynamics.^189^ Also, tools such as TorchMD enable researchers to run simulations using both classical force fields and ML potentials.^190^ A different application of deep learning are convolutional neural networks for reintroducing atomic detail into coarse grained models.^191^ Another approach employed a generative adversarial network to solve the backmapping problem, using as an analogy the image-to-image problem of going from a low to a high resolution image.^192^ Flow matching has also been used to describe coarse-grained force fields which match all-atom ones.^193^ Although progress has been made, these ML force fields are not yet considered mature enough to be used in production simulations and are mostly applied to small molecules or single elements.^194^ In the future, optimization of these force fields is likely to better approximate the interactions between (candidate) drugs and their targets resulting in improved accuracy in virtual screening and MD simulations, but further research is needed.^188^

**Fig. 2:**
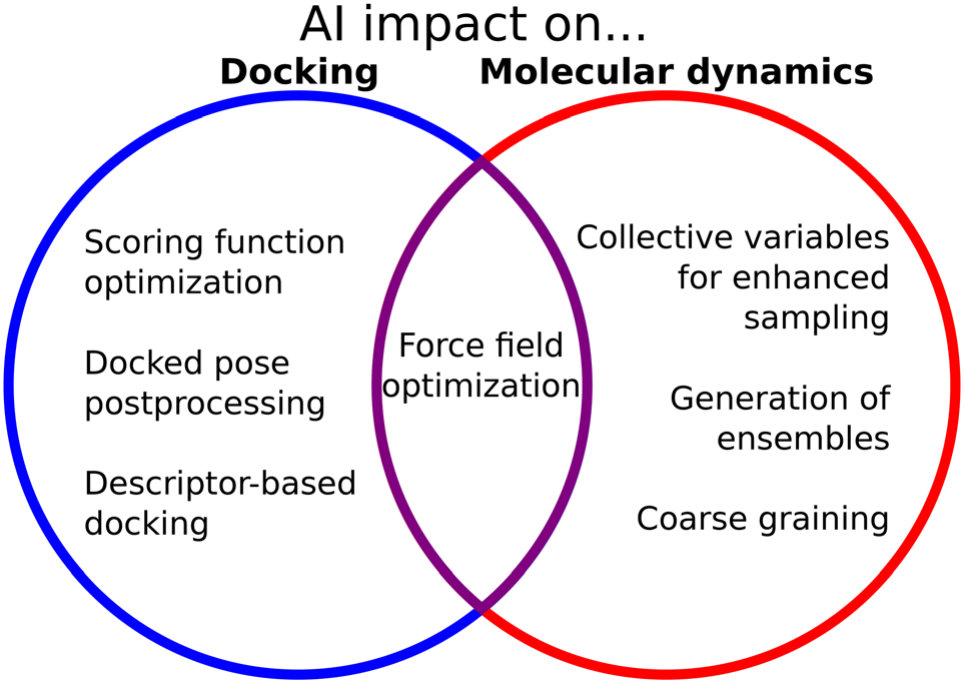
AI influence on docking and MD simulations models and methods.

The interface between biophysical knowledge and ML methods is of utmost importance to expand the capabilities of these models and understand their limitations (Fig. 2). Domain scientists have used and expanded the ML methods, to a great extent. In contrast to CASP14, where AlphaFold2 had a clear dominance, during CASP15 many groups incorporated the ideas from AlphaFold into their pipelines, and the difference in performance was less pronounced.^195^ Structural biologists have also improved ML prediction of proteins by associating different depths of the MSAs with different folds, for example for fold switchers,^96^ disordered proteins,^80,100^ or proteins with different conformations.^97^ Also, knowledge of the biological and biophysical behavior of macromolecules has led scientists to propose ways to incorporate ML into physical methods such as docking or simulations. Janela and Bajorath in particular call for an integration of computational studies into well-planned experimental evaluations to assess the predictive capacity of the different ML methods which are being increasingly proposed.^138^ This is evidence that the interplay between the “hard” ML computer science and the domain application is necessary to find good applications and solve the shortcomings of the original models. It also highlights the importance of the open-source code, which allows scientists to build upon previous work to improve it or find new instances to use it.

## 4. AI applications in drug discovery

An important question is if the recent computational advances can help in hit discovery and/or lead optimization. This question has been asked 20 years ago by Hillisch *et al.* while assessing possible applications of homology modelling in the drug discovery process. Some applications proposed back then included the prediction of binding pockets in homology models of clinically-relevant target proteins, site-directed mutagenesis to (de)sensitize a target to a compound, design of ligands based on the homologous modelled structures, prediction of drug metabolism and toxicity, *etc.*^36^ Modern drug discovery is profiting from AI in several ways, such as studies of structure–activity relationship and data integration.^196^ In contrast, generative drug design does not seem (yet) to be of genuine utility for designing novel molecules in medicinal chemistry campaigns. Thus, for discriminating between hype and real utility it is essential to follow the guidelines formulated by Walters and Murcko for publications of results of generative modeling.^197^ Despite the lack of novel molecules, we hope to see new applications of generative AI in medicinal chemistry. An example is its recent application to scaffold hopping.^198,199^

### 4.1 Describing and quantifying molecules and their interactions

Quantitative structure–activity relationship (QSAR) studies are a natural subject for deep learning integration. Traditionally, linear equations were used to correlate the functional groups and compound properties with the activity observed, but deep learning has also been increasingly used to find relationships between properties and activity.^200^ Unlike “traditional” QSAR, deep QSAR learns the embeddings of the molecules directly, and can also be pretrained with large unlabelled datasets.^201^ An example is provided by Li and Fourches, who trained a general domain model using the ChEMBL database, which then is fine-tuned with target-specific experimental data, to finalize by tranferring these pre-trained model weights to a final QSAR network which is used for the final predictions.^202^ Also related to QSAR is the generation of novel molecules using ML. Initially, *de novo* generation was done by fragment based approaches or evolutionary algorithms.^203,204^ Today, fragment based methods are still in use, having the advantage that the synthetic accessibility can be easily predicted when using rule-based fragment joining.^205,206^ Other generative AI methods include variational autoencoders, generative adversarial networks, flow-based methods, transformer models, diffusion-based, and others, working on different molecular representations such as SMILES or graphs.^207^ An interesting example is provided by Munson *et al.*, who use a variational autoencoder to target two proteins at the same time. They present the problem in an analogous way as networks trained to generate images along different variables, such as age or mood. This allowed them to target pairs of proteins which are together relevant to disease.^208^ Schneider and Clark described several compounds which have been designed *de novo*, albeit sometimes as part of a longer design process usually involving some level of expert input.^209^ To ensure the novelty and to be able to better understand the design process of the AI-generated compounds, Walters and Murcko have called for transparent reporting of the datasets used for training, showing the most similar molecule in the training set, and evaluating the molecules with the same criteria as those generated by medicinal chemists.^197^ Nonetheless, new AI-generated compounds have already entered clinical trials as treatments against diverse illnesses including atopic dermatitis, neurofibromatosis type 2, and others.^210^

The way in which small molecules are digitally represented is essential for QSAR studies, and also cheminformatics. Initially, “traditional”, or “bespoke” descriptors, as called by McGibbon *et al.*, were the main types of molecular representations.^211^ The advent of ML in cheminformatics means that new representations can now be “learned” from data. The input representation and the type of ML method used for embedding the represented molecule determine the type of encoding needed.^212^ McGibbon *et al.* describe three main types of learned representations. First, convolutional encodings have a high tolerance for many different inputs, but their main limitation is the lack of rotation-invariance. Second, graph encodings represent molecules and their features as a graph, and can be used by a variety of neural network architectures. Finally, the string encodings are traditionally used with transformer architectures.^211^ An example of a string-based representation designed specifically for ML-based methods is SELFIES.^213^

Molecular descriptors are representations which encode the physicochemical information of the molecule. They can be derived from experimental data, such as the solubility or the octanol/water partition coefficient, or be theoretically defined. Theoretical descriptors can also vary in the level of abstraction, ranging from adimensional descriptors such as molecular weight and heavy atom number, up to four-dimensional descriptors encoding the interactions with binding partners.^214^ Fingerprints are a type of representation based on encoding descriptors into a vector.^215^ The importance of accurate descriptors was highlighted by van Tilborg *et al.*, who showed that SAR predictors based on molecular descriptors outperformed deep learning models based on SMILES or graphs.^216^ Therefore, the use of learned descriptors should be carefully considered, for example Capecchi *et al.* describe a molecular fingerprint based on substructures which performs well, without the need of a ML-based encoding.^217^ It is important to note that in general the prediction of bio-activity data (*e.g.*, binding potency for the target) is a more challenging task than learning physicochemical properties (*e.g.*, aqueous solubility) or ADME (absorption, distribution, metabolism, and excretion) properties. Furthermore, bio-activity data is usually sparser which is a strong limitation for ML methods.

### 4.2 AI beyond descriptors

AI also has a role in helping medicinal chemists plan their synthetic activities. Traditional retrosynthesis prediction relies heavily in chemical knowledge to set the rules of reactions. Language models can exploit the analogy between language and organic chemistry^218^ to predict synthetic precursors.^219^ Apart from predicting the reactions themselves, it would be valuable to predict their yield. To this effect, Schwaller *et al.* built a transformer model to predict reaction yields based on SMILES representations. They have achieved this by combining a reaction SMILES encoder with a reaction regression to predict the yield, and speculate this could be applied to other regression tasks such as activation energies.^220^ In other study, Schwaller *et al.* tackled the problem of reaction classification, also using a transformer.^221^ Nevertheless, it is important to note that reaction fingerprinting based on *k*-nearest neighbors can achieve comparable accuracy in reaction classification and yield prediction with much less complexity.^222^

Other applications of deep learning in drug discovery include for example drug repurposing. An example comes from Zhang *et al.*, who used a transformer based on SMILES-protein sequence pairs to predict commercially available antiviral drugs which could be used against SARS-CoV-2.^223^ A similar study was performed by Beck *et al.*^224^ A completely different approach was taken by Yan *et al.*, who prompted ChatGPT with the task of proposing approved drugs which could be useful against Alzheimer's disease. They theorized that the model's ability to efficiently parse literature could be a reason for the plausibility of the suggestions it generated.^225^ These are examples of what Vincent *et al.* describe as one category of ML studies in the area of phenotypic drug discovery. In this case, pharmacology data from other studies is transferred and used to predict new scaffolds for a given disease. The other category of studies is those that use phenotypical data, for example training them directly on data on cellular perturbations or gene expression changes.^226^ Returning to target-based drug discovery, ML can be a useful help in finding new targets. ML models can be a useful tool for drug target prediction, meaning instead of finding a good binder for a specific target, existing molecules can be screened and their protein target predicted.^227^ An example of drug–target interaction prediction is MolTrans, which based on a transformer classifier, predicts whether a drug–protein pair will interact.^228^ A coupling of these techniques could be applied also in basic research. For example, given phenotypical data, drug–target interaction prediction could be used to find the mode of action of a drug.

Another active line of research concerns foundation models, which are usually large language models which are pretrained on large amounts of data to be later finetuned for a specific task.^229^ These are similar to transfer learning, which is generally applied in drug discovery.^230^ An example of transfer learning was presented by Tysinger *et al.*, where the authors pretrain a transformer model based on pairs of bioactive molecules from ChEMBL, and then use it to predict new molecules using known hits as input.^231^ Unfortunately, this paper does not include any perspective, *i.e.*, experimental validation of the transformer model. A foundation model was used by Chenthamarakshan *et al.* to predict new binders using pretrained molecular and protein representations to classify molecules as binders or not, while also taking into account off target effects and synthesizability.^232^ Chang and Ye presented a bidirectional model linking SMILES and property prediction.^233^ Finally, a concept that is recently expanding is that of digital twins, where systems of various complexities, cells for example, are represented virtually, enabling *in silico* experiments to be performed on them. A dialogue is then set between the digital twin and the experimental data, such that the twin can be fine tuned with the experimental data, and the predictions used to inform further experiments.^234^

Deep learning and related methods can have a strong impact in drug discovery, for example in aiding the sampling of novel chemical space and the virtual screening of these myriad new compounds, for generating quantum mechanics-level descriptors of molecular interactions, and to accelerate virtual screening.^201^ In a time when target-based drug discovery has been described as inefficient, ML techniques could open the door to integrative data modelling which can yield not only the binding affinity to a single target, but also a prediction of the phenotypical effect of the screened molecules.^235,236^ Challenges remain such as data curation and availability, or the increasing complexity in the types of data available. Additionally, molecular generation models need to be validated to ensure their output is sensible.^237^ Still, ML methods could accelerate the drug discovery process not only by finding molecules that bind a target with high affinity, but also yield the desired effect based on omics data, phenotypical observation, selectivity prediction, or pharmacokinetics. Furthermore, test cycles could be reduced using predicted data, or the synthesis of new compounds made easier with retrosynthesis prediction tools.^238^ In the end, the most important aspect will be to find a balance between the outputs of the ML models, and the human creativity of the medicinal chemists and structural bioinformaticians applying them.^239^ AI applications in biology will continue to expand these coming years, in drug discovery and other areas,^240^ but although several steps of the process have profited from data-driven augmentation, for now, human intervention remains essential.^241^

## 5. Is AI really what we need?

### 5.1 AI imposes a burden on resources

The ML explosion of the past few years has provided potential improvements in different stages of the drug discovery process, but it is also accompanied by some concerning trends following the wide application of AI. An usually forgotten factor when using ML models, and indeed also when running simulations,^242,243^ is the energy consumption involved in the training and use of the models. Recent reports show that the “AI boom” of the past years is already threatening the climate goals of tech companies.^244,245^ The International Energy Agency estimates that data centers, cryptocurrencies and AI have consumed a 2% of the total energy used in 2022. The projected growth of these sectors until 2026 means their electricity needs will be equivalent to the energy consumption of Germany.^246^ The different sources of electricity used in the places where AI models are trained, the cost (economic and environmental) of manufacturing the needed devices, and the diverse infrastructures where they are housed add increased complexity to the calculation of the environmental impact of AI models. The electricity and carbon cost of training one iteration of a model, *e.g.*, GPT-3 (around 1.2 GWh and 588 tons of CO_2_ equivalents) seems negligible on its own,^247^ but adds up quickly when considering the number of training runs needed to obtain a final version and the current explosion in the number of AI methods. Thus frameworks for quantifying and regulating the emissions due to AI (training and inference) are urgently needed.^248^ Water is another equally important natural resource which is put under pressure for training and deploying AI systems. Water is used in the cooling towers of power plants generating electricity for AI, but also cooling systems of data centers themselves, and also during the manufacturing of computing infrastructure (chips). A recent estimate suggests that the training of the GPT-3 model requires on the order of 700 000 liters of water.^249^ Obtaining accurate AI models while reducing the carbon output can be achieved through careful selection of the model used and has already been done by scientists.^250^ Although individual action on its own is not enough to stop the detrimental advance of climate change,^251^ we should as scientists be conscious of the impacts of our development and use of AI, and push for new paradigms in the deployment of such systems.^252,253^ Using a combination of metrics such as efficiency and interpretability, in addition to accuracy, could be an answer to have better-designed and -implemented deep learning models, depending on fewer parameters, and therefore also reducing their economic and environmental cost.^254^

**Fig. 3:**
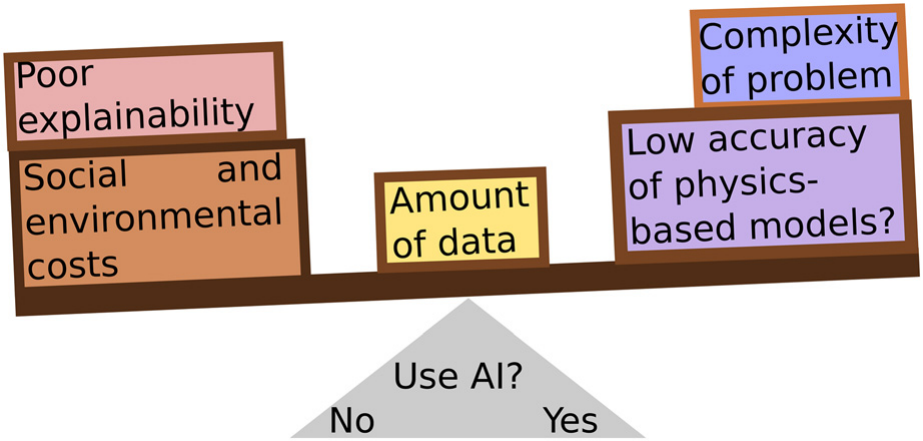
The decision whether to deploy an AI model must be a careful consideration of several factors: can the problem be solved with simpler, *e.g.*, less data-hungry, methods? Is there enough data to train an AI model? Is explainability necessary for understanding the problem? Does the problem justify the costs of training and deploying an AI model?

Careful consideration must be taken when choosing to use an AI solution in drug discovery, and indeed in any case. The goal of any scientific application should be to solve a problem in the most efficient way, not necessarily using the most advanced model (Fig. 3). Simpler models can be deployed for appropriate tasks, such as fingerprinting and regression for chemical properties,^217^ conformer generation,^255,256^ docking,^257,258^ molecular descriptors,^259^ reaction classification and yield prediction,^222^ or others. Meanwhile, AI can be reserved to those tasks that merit the cost of deploying it. As scientists, we should take care to consider the environmental, social, and ethical costs of AI systems while making this choice.

### 5.2 The knowledge of AI is becoming concentrated

Another concerning tendency that is manifesting itself, not only in biology, is the privatization of science. Deep learning systems, like AlphaFold,^65^ make use of public datasets such as the Protein Data Bank for training.^260^ This data has been collected over many decades and financed mostly through public money. Indeed the replacement cost of the PDB has been estimated at around USD 20 billion.^261^ Nevertheless, AlphaFold3,^66^ the latest iteration of the greatly successful deep learning protein structure prediction model, has been published without making its code freely available, angering scientists.^262^ This decision even went against the editorial policy of its journal, which justified itself by claiming AlphaFold3 was privately funded and the service is still open and publicly available.^263^ Only during revision of this manuscript, *i.e.*, about six months after its initial release, the code of AlphaFold3 was made public, with strong licensing restrictions. AlphaFold3 was developed by Alphabet's subsidiaries DeepMind and Isomorphic Labs, the latter of which will use it for its rational drug design campaigns.^66,264^ Indeed, Fernández Pinto argues, this is a common characteristic of Open Science, where data generation is financed by the public and shared openly, but the private actors are not compelled to maintain the same standards and end profiting from the system.^265^ Rikap shows how the assetization of knowledge and data to generate intellectual monopolies is a common strategy of tech giants.^266^ Preventing intellectual monopolies could allow diverse smaller companies access and adopt the AI drug discovery workflows at a more competitive rate, instead of concentrating the technology on the tech giants with the money and IP to deploy them. A more critical standpoint might even argue that it is valid to oppose to profits being made from publicly sourced science. From the scientific point of view, the monopolization and platformization of AI research makes it more difficult to benchmark and improve these methods. Therefore, safeguards and regulations must be implemented to ensure that knowledge generated by AI is kept open and public, and to prevent that public infrastructures end being abused and profited from by private companies.^267^ A framework moving towards this goal is needed and work on it is underway, to ensure not only public and open AI, but also that the society that contributed to the models can benefit from it.^268,269^

## 6. The future of ML in biology

What are the outstanding issues and next research directions in protein structure prediction and design? Current ML methods for protein structure prediction are trained on secondary data, *i.e.*, data derived usually by fitting from the raw (primary) data. Networks like AlphaFold are trained on the protein structures available on the PDB, to obtain a probabilistic model of protein structure, and generate (a set of) single structures as output. The prediction of a single structure has been proposed as the current main limitation of these methods.^84^ Nevertheless, the structures employed for training have also been fitted onto an electronic density (from X-ray crystallography or CryoEM), which is also modelled from the collected data. So far, researchers have used deep learning methods to reconstruct the protein structure into CryoEM maps,^270–272^ predict flexibility,^273^ or to solve the phase problem in X-ray crystallography for short peptide sequences.^274^ Zhong *et al.* take an interesting direction, using variational autoencoders that encode CryoEM images and decode density maps, taking heterogeneity into account.^275^ A similar method has been proposed by Rosenbaum *et al.*^276^ The use of ML methods in CryoEM has expanded a lot and will probably continue to expand itself in the coming years.

To date, no method for protein structure prediction seems to be based on anything but static structures. Therefore, a possible paradigm shift could be to stop training the networks on the atomic coordinates of a single structure, and instead try to assign a probability distribution to their electronic densities, on which the deep neural networks can then be trained on. A possible difficulty arising from this is the (nearly impossible) assignment of continuous densities to the electronic cloud of single atoms, but this could be modelled based on the (constructed) structures which have been deposited. Nevertheless, and continuing with the translation from computational linguistics to biology, this could be seen as an analogous problem to the recognition of handwritten cursive text in computer vision.^277–279^ Indeed, recent work has been done on generating the “MNIST of aminoacids” dataset, which could be an initial step towards assigning the continuous electron density to discrete residues.^280^ Using this type of data could be a new way to tackle the lack of conformational ensembles from AlphaFold and related models.^63^ Another possibility is to use raw data from NMR to augment ML networks. As discussed earlier, coevolutionary restraints from the MSAs applied in DL-based structure prediction work analogously to NMR structure determination using distance restraints. NMR data has been used to enhance the predictions of AlphaFold and obtain structures that comply with the experimental restraints,^281^ or to assess the AlphaFold predictions.^79,282^ However, training a network directly on NMR data has only been attempted for small molecules.^283^ Of course, a problem which could arise comes from the fact that NMR experiments usually yield very few restraints, which are sometimes redundant. Still, an interesting avenue of research would be to incorporate different experimental data into the training of the deep learning model such that it can use the different structures, CryoEM electron densities, and NMR restraints to predict ensembles of structures.

Finally, something we consider as extremely interesting would be to try to abstract the physical rules of protein folding, and indeed biomolecular interaction, from deep learning models for structure prediction. As discussed above, decades of research were invested into *ab initio* protein structure prediction with moderate success. It has been widely established that folding follows physical “rules”. At folding conditions, the native conformation is the thermodynamically most stable state, located at the bottom of the free energy funnel.^284^ Is it possible that the physical models used for protein structure prediction until now were too simple to capture all the interactions needed to determine the three-dimensional conformation? An advantage of AI-based methods is their ability to learn representations of, or embed, complex data. If AlphaFold has learnt an energy function for protein structure, as proposed by some authors,^98,99^ there should be a way to extract this information from the model to try to understand the physical interactions which govern protein structure and folding. While this claim has been disputed,^94,96^ it is undeniable that the model predicts globular proteins with high accuracy. One of the newer trends in AI research is explainable AI (xAI).^285^ There is no exact definition of xAI, but it can be understood as the set of methodologies which help users understand and believe the models and predictions from AI.^286^ It would be interesting to apply the xAI framework, for example by examining the attention mechanism in AlphaFold, to try to understand how it predicts inter-residue interactions. Such an approach would offer a bridge between the “black box” approach of deep learning, and physics-based methods. The high-dimensional abstraction of the AI model could be used to better capture the interactions that govern protein folding and thus improve *ab initio* methods. A pitfall for this would be if AlphaFold has not learned the energy function of protein structure but rather it bases its predictions in memorization,^96^ if the prediction is actually relating MSAs to structure instead of the sequence,^71^ and indeed if the MSAs and the coevolutionary information they encode are not enough to predict different conformations. More research is needed to understand if AlphaFold and related methods can actually be interpreted and used to understand the physics behind protein folding.

## 7. Conclusions

We have reviewed the recent progresses of ML tools and their influence on protein structure prediction and computer-aided drug design. A long journey from the determination of the first crystal structure of a protein (myoglobin, more than 60 years ago) has culminated into half of the 2024 Nobel prize in chemistry which was awarded to the main developers of the deep learning programs for protein structure prediction. It is evident that these ML tools have predictive ability mainly because they are trained on the more than 200 000 experimentally determined structures of proteins. In turn, such a rich data set of protein structures exists because of impressive progress in the (mainly bacterial) production of pure proteins and the hard work of many research groups most of which are affiliated with not-for-profit institutions. The interplay and synergism between physics and AI are expected to grow in the near future. The data-driven methods for modeling protein structures based on sequence homology have evolved into very powerful deep learning platforms. At the same time, the knowledge-based docking functions are being enhanced by using ML methods. Even force fields are being improved thanks to the large availability of data which can be exploited by ML models. The rich intersection between scientific disciplines such as the integration of language models into biology, or the physical inspiration of AI,^287^ are opening new ways to tackle the shortcomings of current ML models. This exchange needs to be promoted to obtain better systems for protein structure prediction, docking of small molecules, or generation of ensembles. These advances did not happen from one day to the other, and it is important to recognize all the scientific progresses which enabled these technologies to be implemented and which are often forgotten.

Another important factor to consider is the cost of ML from the point of view of the natural resources needed to train and deploy it. This consideration should be central to decide whether it is justifiable to use an ML system in research, or if there are simpler (physics-based) solutions that can yield comparable and more explainable results with less energy consumption.

We should also be aware of the tendency for ML models to become services which scientists can use but have no full control over. This is exemplified by the most recent AlphaFold3. The emergence of “Science as a Service” already is hindering our understanding of the working of AlphaFold3 and other deep learning models. Therefore we should fight for control and access to these ML models which would not exist without the decades of work of generations of scientists supported mainly by public funding. As an example, during the past decade the research group of the senior author of this review has released in the PDB database more than 300 high-resolution crystal structures of proteins of pharmacological interest in complex with small molecule–ligands. Interestingly, most of these 300+ ligands were identified by high-throughput docking and force field-based binding energy evaluation.^110,288–290^ Hence, we can state that physics-based docking, at least in the Caflisch group, and more generally protein X-ray crystallography have contributed substantially to the data used for training AlphaFold3. Thus, we should be prepared to discuss and set the rules of the game for open access to ML tools trained on open access data.^291^

Several challenges lie ahead on the road. Successful prediction of single protein structures is possible efficiently and routinely, but the generation of conformational ensembles requires additional effort. The most-widely used methods for protein structure prediction, and AI models in general, often lack explainability, making it difficult to understand their workings. For hit identification, physics based methods have been used in (high-throughput) docking programs for long, but their scoring functions can be further improved for reducing the number of false positives and thus increase the hit rates. Concerning hit to lead optimization, it has been noted that ML tools, *e.g.*, generative modeling, are useful for interpolating within a known chemical series but are not able to extrapolate to new chemical matter.^197,292,293^ The latter seems possible (at least for some chemical series and protein targets) by MD free energy-calculations which have improved substantially in accuracy thanks to continuous optimization of force fields (during the past decade particularly for small molecules) and faster hardware. Physics-based models use analytical energy functions (force fields) which achieve high accuracy, speed, and extensive coverage of chemical space by employing a fraction of the parameteres used by ML models.^294^ In conclusion, ML methods have evolved by taking advantage of decades of previous research in computational and structural biology. Now it is up to us to promote the physics-AI dialogue to successfully combine ML tools and physics-based methods for designing new drugs.

## Data availability

No primary research results, software or code have been included and no new data were generated or analyzed as part of this review.

## Author contributions

PV and AC wrote this review.

## Conflicts of interest

There are no conflicts to declare.
